# Mixing of immiscible polymers using nanoporous coordination templates

**DOI:** 10.1038/ncomms8473

**Published:** 2015-07-01

**Authors:** Takashi Uemura, Tetsuya Kaseda, Yotaro Sasaki, Munehiro Inukai, Takaaki Toriyama, Atsushi Takahara, Hiroshi Jinnai, Susumu Kitagawa

**Affiliations:** 1 Department of Synthetic Chemistry and Biological Chemistry, Graduate School of Engineering, Kyoto University, Katsura, Nishikyo-ku, Kyoto 615-8510, Japan; 2 CREST, Japan Science and Technology Agency (JST), 4-1-8 Honcho, Kawaguchi, Saitama 332-0012, Japan; 3 Institute for Integrated Cell-Material Sciences (iCeMS), Kyoto University, Yoshida, Sakyo-ku, Kyoto 606-8501, Japan; 4 Institute of Materials Chemistry and Engineering (IMCE), Kyushu University, 744, Motooka, Nishi-ku, Fukuoka 819-0395, Japan; 5 Institute of Multidisciplinary Research for Advanced Materials (IMRAM), Tohoku University, 2-1-1, Katahira, Aoba-ku, Sendai 980-8577, Japan

## Abstract

The establishment of methodologies for the mixing of immiscible substances is highly desirable to facilitate the development of fundamental science and materials technology. Herein we describe a new protocol for the compatibilization of immiscible polymers at the molecular level using porous coordination polymers (PCPs) as removable templates. In this process, the typical immiscible polymer pair of polystyrene (PSt) and poly(methyl methacrylate) (PMMA) was prepared via the successive homopolymerizations of their monomers in a PCP to distribute the polymers inside the PCP particles. Subsequent dissolution of the PCP frameworks in a chelator solution affords a PSt/PMMA blend that is homogeneous in the range of several nanometers. Due to the unusual compatibilization, the thermal properties of the polymer blend are remarkably improved compared with the conventional solvent-cast blend. This method is also applicable to the compatibilization of PSt and polyacrylonitrile, which have very different solubility parameters.

## Abstract (web summary)

The intimate mixing of immiscible polymers is desirable both to aid understanding of the fundamental science and for the development of new materials. Here, the authors successfully compatibilize polystyrene and poly(methyl methacrylate) using a porous coordination polymer as a removable template.

## Graphical abstract

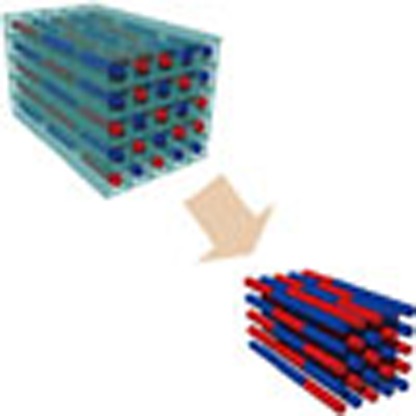

Homogeneous mixing of chemical substances is crucial in chemistry, materials and technology. For example, a solute needs high compatibility with a solvent to afford a homogeneous solution. Cocrystallization of two or more components leads to create supermolecules, which is relevant to production of pharmaceuticals and new framework materials^1^,^2^. Among the mixing systems in chemistry, much scientific and commercial attention has been devoted to compatibilization of macromolecules in efforts to produce new materials with tailored properties^3^,^4^. However, different from low-molecular-weight compounds, the entropy of mixing for macromolecules and colloidal particles is inherently very low; therefore, in most cases, the mixing of two or more polymers results in phase separation on the macroscopic scale^5^. This fatal problem poses a significant barrier to producing many scientifically and technologically relevant homogeneous blends, including interface in adherend–adherate, heterojunction in organic photovoltaics and cells in display and battery. Homogeneous mixing of chemically immiscible substances would allow synergistic tunability and significant enhancement of the resulting material's potential functions, which could realize new opportunities that would otherwise be impossible to achieve with the individual components on their own. Here, we disclose a new methodology that allows immiscible polymers to be mixed at the molecular level by facilitating polymerization within porous coordination matrices followed by dissolution of the hosts to liberate the polymer blend (Fig. 1). In this system, the porous coordination templates can be easily and effectively removed using an aqueous chelator solution, resulting in the intimate mixing of polymer pairs that have never been before compatibilized molecularly.

One way to circumvent the macroscopic phase separation of polymers is to use well-defined block copolymers, enabling nanoscopic structural control of the segregated polymer domains^6^,^7^,^8^. However, the molecular-level compatibilization of homopolymers remains a great challenge. Chemical compatibilization of polymer blends is conducted via the introduction of reactive groups in precursor polymers to connect individual polymer chains covalently^9^,^10^. However, this type of chemical reaction induces inevitable modifications of structures, resulting in a change in processability and polymer properties. In contrast, a physical approach can provide polymer blends without changing the structure and composition of the individual polymers. Tremendous efforts have been made to minimize interfacial tension between polymers using a variety of compatibilizers^11^,^12^,^13^. Although the domain size of phase separation can be reduced, homogeneous mixing in the range of a few nanometers cannot be accomplished, and the resulting polymer blend is contaminated with another chemical substance (compatibilizer). To overcome the limitations of the existing approaches, several strategies have recently appeared to prepare polymer blends using supramolecular complexation, polymer nanoparticles, fluid interfaces or electrospinning^14^,^15^,^16^,^17^,^18^; however, it remains difficult to improve versatility, mass-productivity and/or compatibility. Hence, the search for an easy and efficient strategy to achieve the perfect mixing of polymers at the molecular level continues.

Thermodynamic non-equilibrium approaches are often useful for controlling the arrangement of a polymer assembly^19^,^20^. This is due to the slow dynamics of polymer chains, which causes the kinetic manipulation of polymers to give unique polymer architectures with unconventional assembly and morphology. One kinetic approach involves the utilization of nanoporous templates, including zeolites and organic cages, resulting in many specific polymer conformations and assemblies^21^,^22^,^23^,^24^,^25^. These can be created via accommodation of polymers inside the nanopores followed by the destruction of the nanoporous hosts. However, removal of these hosts consisting of covalent bonds requires relatively harsh conditions, often leaving the residues of host matrices and/or resulting in unfavourable rearrangements of polymers, which may preclude the formation of blends at the molecular level.

Recently, porous coordination polymers (PCPs) or metal organic frameworks composed of transition metal ions and bridging organic ligands have emerged as new porous materials with a wide range of potential applications, such as gas storage, separation, catalysis and molecular release^26^,^27^,^28^,^29^,^30^. The pore structures of PCPs are highly tunable, based on the combination of components, and can be readily decomposed under mild conditions, making them different from the conventional porous materials (zeolites, activated carbons, organic cages and so on). A variety of chemicals and materials, such as organic compounds, inorganic particles and biomolecules, can be introduced in PCPs, affording functional host–guest nanohybrids. In particular, an increasing number of papers report on the preparation or incorporation of polymers within the nanochannels of PCPs in an effort to control the structures, assemblies and properties of the polymers^31^,^32^,^33^,^34^,^35^. These encapsulated polymers can be easily isolated by dissolution of the supramolecular host frameworks in mild chelating solutions^31^,^32^,^35^. Thus, our strategy to achieve polymer mixing involves using PCPs as templates (Fig. 1). Many different combinations of a variety of polymers can be mixed inside the PCPs, in which the nanochannels can accommodate a random distribution of polymer chains. Subsequent removal of the host PCPs results in the polymer blend, thereby ensuring the intimate mixing state of the polymers. This strategy holds several advantages for the preparation of polymer alloys: no need for modification of the polymer structure, high versatility of applicable polymers and the possibility of achieving the molecular-level compatibilization without the requirement for any additives. A recent report showed that the simple mixing of PCP (ZIF-8) nanoparticles in polymer blends exhibited compatibilizing effects on controlling the domain size in the submicrometer region^36^.

Here, in contrast, our protocol demonstrates that immiscible polymers can be completely compatibilized in the single nanometer range even after the removal of PCPs. To test this approach, we employed polystyrene (PSt) and poly(methyl methacrylate) (PMMA), the most typical pair of immiscible polymers, despite their close solubility parameters (solubility parameters of PSt and PMMA are 8.5–9.3 and 9.1–12.8 (cal cm^−3^)^0.5^, respectively).^37^ To our knowledge, there has as yet been no successful attempts to demonstrate the homogeneous compatibilization of PSt and PMMA on the single nanometer scale.

## Results

### Preparation of PSt/PMMA blend

Sequential synthesis of PSt and PMMA was carried out in [Zn_2_(bdc)_2_(dabco)]_*n*_ (**1**: bdc=1,4-benzenedicarboxylate, dabco=1,4-diazabicyclo[2.2.2]octane), because this PCP can produce many vinyl polymers in radical process and can be easily digested in a mild condition^35^,^38^. The St monomer and radical initiator were introduced into the channels of **1** by immersion of the PCP particles in St, followed by the removal of excess St external to the host crystals by evacuation. Then, the host-monomer adduct was heated at 70 °C to induce polymerization. In this polymerization system, MeOH was added to quench the polymerization, and the residual monomers were removed by washing with MeOH. Evacuation of the product (**1**⊃PSt) at 100 °C could remove MeOH in the pores, regenerating the porosity that could then be used for the synthesis of a second polymer, PMMA. After filling the remaining pores with MMA, we performed the polymerization in the same manner to obtain a PCP composite containing PSt and PMMA (**1**⊃PSt/PMMA). Scanning electron microscopy (SEM) of **1**⊃PSt/PMMA showed the maintenance of the morphology of the host crystals after the polymerization, which confirmed that polymerizations proceeded entirely within the nanochannels of **1** (Supplementary Fig. 1). No change in the peak patterns was observed in the X-ray powder diffraction (XRPD) profiles of **1** during the polymerization of both monomers, indicating that the nanochannel structure was retained on the inclusion of monomers and their polymerizations (Supplementary Fig. 2). Porosities of **1** and the composites were examined by nitrogen adsorption measurements at 77 K (Supplementary Fig. 3). A decrease in the adsorption amount of **1**⊃PSt compared with that of **1** alone confirmed the encapsulation of PSt chains in the channels of **1**. A further decrease in the adsorption amount of **1**⊃PSt/PMMA was consistent with the additional introduction of PMMA in the residual pores of **1**⊃PSt.

Quantitative isolation of the polymers from **1**⊃PSt/PMMA was performed by dissolution of the PCP framework in 0.5 M aqueous sodium ethylenediaminetetraacetate (Na-EDTA) solution^35^,^38^. SEM images of the product indicated cubic polymer particles, whose morphology was similar to that of **1**⊃PSt/PMMA, despite the complete removal of the host framework (Supplementary Fig. 1). Furthermore, XRD patterns of the isolated polymers did not show any peaks corresponding to residues of **1** (Supplementary Fig. 2). The amount of Zn in the polymer product was negligible (<0.5 wt %), as confirmed by X-ray fluorescence spectroscopy. The formation of PSt and PMMA homopolymers, with molecular weights of several tens of thousands, in the pores of **1** was fully analysed by infrared, nuclear magnetic resonance (NMR) and gel permeation chromatography (Supplementary Figs 4–6). Stereoregularities of the polymers were atactic, and close to those of PSt and PMMA prepared under free radical conditions, as reported previously^38^. In the ^1^H NMR analysis, the molar ratio of PSt in the polymer blend could be determined from the integrations of the signals for PSt and PMMA (Supplementary Fig. 5). This polymer ratio in the product can be rationally tailored by changing the polymerization time of the monomers (Methods; Supplementary Fig. 7). In general, the molar ratio of PSt in the blend increases with increasing polymerization time of St. In this work, we used an approximately equimolar mixture of PSt and PMMA for further analyses, unless otherwise noted.

### Characterizations of the polymer blend

**Figure 2:**
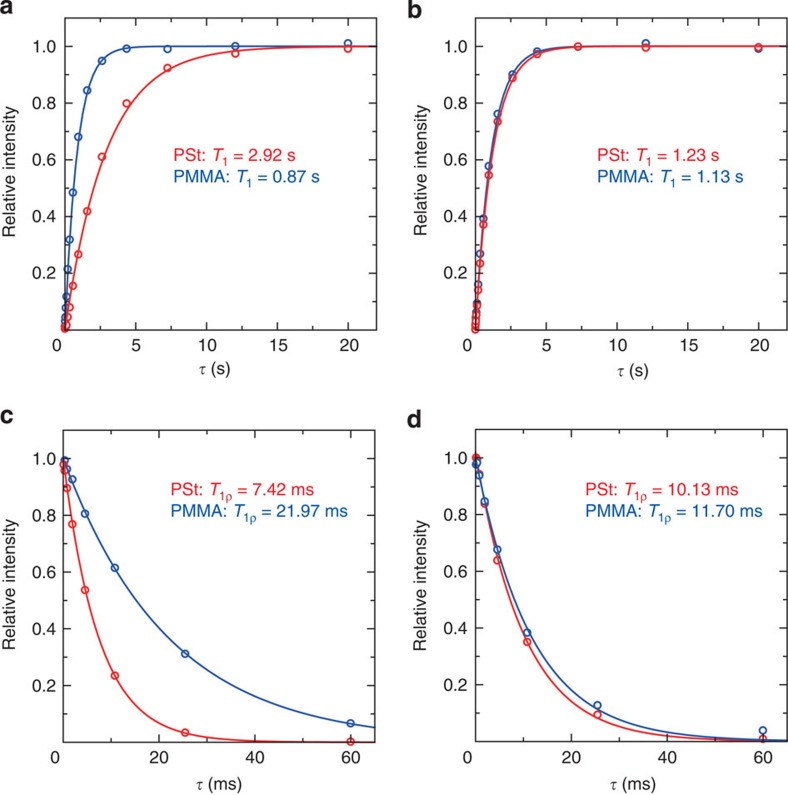
Spin-lattice relaxation time measurements of PSt/PMMA blends. The observed *T*_1_ recovery curves for aromatic carbons of PSt (red) and carbonyl carbon of PMMA (blue) in (**a**) CHCl_3_-cast blend and (**b**) blend isolated from **1**⊃PSt/PMMA. The observed *T*_1ρ_ decay curves for aromatic carbons of PSt (red) and carbonyl carbon of PMMA (blue) in (**c**) CHCl_3_-cast blend and (**d**) blend isolated from **1**⊃PSt/PMMA.

The compatibility of the PSt and PMMA blend can be investigated by ^1^H spin-lattice relaxation time measurements using solid-state ^13^C cross-polarization magic-angle spinning (CPMAS) NMR experiments (Supplementary Fig. 8). Analysis of the ^1^H relaxation time can provide useful insight into the domain size, phase diagram and compositional fluctuation of polymer blends^39^,^40^. In a conventional polymer blend with macroscopic phase separation, each polymer domain has different characteristic ^1^H relaxation times. However, polymer blends mixed on the nanometer scale show the same relaxation times for both polymers because ^1^H spin diffusion occurs among all protons in the blends. In fact, CHCl_3_-cast blend of PSt and PMMA (PSt/PMMA=1:1) showed two distinct ^1^H spin-lattice relaxation times in the laboratory frame (*T*_1_) corresponding to the two polymer components (Fig. 2a). In contrast, closely similar *T*_1_ values were observed for PSt and PMMA in our sample, indicating that the two polymers were mixed at least within several tens of nanometers (Fig. 2b). To examine the homogeneity of the blend in the range of a few nanometers, we further measured the spin-lattice ^1^H relaxation time in the rotating frame (*T*_1ρ_) using ^13^C CPMAS NMR spectra. In this analysis, quite different *T*_1ρ_ values for PSt and PMMA confirmed the clear phase separation in the solvent-cast sample (Fig. 2c). However, it was evident that the polymer blend obtained from **1** had almost the same *T*_1ρ_ values for PSt and PMMA, suggesting their intimate compatibilization at the molecular level (Fig. 2d).

**Figure 3:**
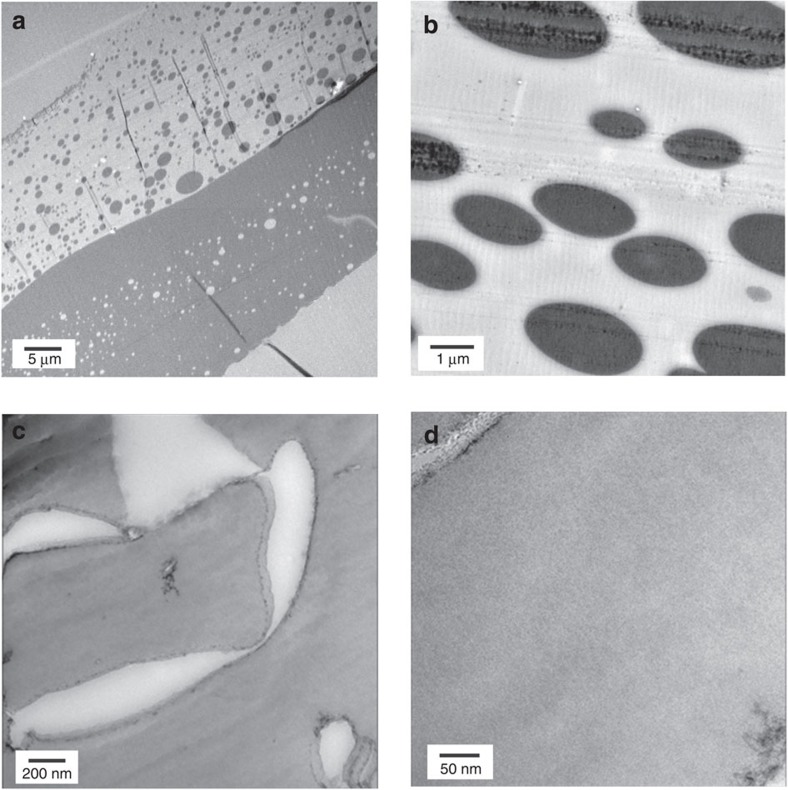
Transmission electron microscopy of PSt/PMMA blends. TEM images of PSt/PMMA blend (**a**,**b**) obtained by casting from CHCl_3_ solution and (**c**,**d**) isolated from **1**⊃PSt/PMMA. RuO_4_ was used for staining the PSt domains in both samples. (**a**) Macroscopically phase-separated bilayer structure with sea-island morphology (dark and bright areas correspond to PSt and PMMA domains, respectively) was seen in the image of equimolar film of PSt and PMMA obtained from CHCl_3_ casting. (**b**) The sea-island phase-separated structure was observed when increasing the ratio of PMMA in the cast blend (PSt/PMMA=35:65). (**c**) PSt/PMMA blend isolated from **1** did not contain obvious polymer domains inside the particles. (**d**) No apparent contrast was observed even in the highly magnified image of the PSt/PMMA blend obtained using **1**, indicating molecular-level mixing of PSt and PMMA in the blend sample.

Direct visualization is the most powerful method to prove the compatibility of the polymers. We therefore analysed the nanoscale morphology of the polymer blends using transmission electron microscopy (TEM). TEM micrographs of CHCl_3_-cast PSt/PMMA blends are shown in Fig. 3a,b. Note that this cast sample was first embedded into epoxy resin and then microtomed to thin sections, which were then stained with RuO_4_ vapour to selectively stain the PSt-rich domains. In the TEM images, an equimolar blended sample exhibited a macroscopically segregated structure with bilayered PSt- and PMMA-rich phases, where island domains of PSt were observed in the PMMA phase and *vice versa* (Fig. 3a)^41^. The sea-island morphology was predominantly observed in the cast blend with the unequal polymer ratios (Fig. 3b)^41^. A polymer blend isolated from **1** was likewise subjected to TEM observations. The sample was exposed to RuO_4_ vapour before it was embedded into the epoxy. Exposure to RuO_4_ vapour is necessary not only to stain the PSt domains but also to emphasize the edge of the sample. Because the densities of the blend and the epoxy may be similar, the edge of the sample may not be discerned without this pre-staining protocol. In fact, RuO_4_ aggregates were found at the surface of the exterior of the particles (Fig. 3c). After microtoming the epoxy-embedded particles to thin section, staining with RuO_4_ was repeated prior to the TEM observations. Interestingly, the TEM image of the PSt/PMMA blend isolated from **1** displayed no apparent contrast on the single nanometer scale inside the blend sample (Fig. 3c,d). This clearly demonstrated the homogeneous mixing of the polymers at the molecular level, in marked contrast to the solvent-cast sample (Fig. 3a,b). Note that treatment of the polymer blend with CHCl_3_ and subsequent casting gave macroscopically phase-separated polymers (Supplementary Fig. 9). Thus, all these results indicated that the use of a PCP template could successfully allow molecular-level mixing of a typical immiscible polymer pair, PSt and PMMA.

### Stability of the polymer blend

In the blending system under discussion, the morphology of the polymer particles from **1** was almost the same as that of the host PCP (Supplementary Figs 1 and 10). Previous papers have reported that the morphological maintenance results from the low dynamics of polymers at room temperature, where the glass transition temperatures (*T*_g_s) of PSt and PMMA are 105 °C and 120 °C, respectively^21^,^35^. This should be crucial to attain the intimate compatibilization of the polymers, because the molecularly mixed state of PSt and PMMA through the medium of pore walls of **1** could be substantially preserved during the isolation process due to the inability of the polymers to be self-organized at room temperature. It was interesting to note that the kinetically trapped PSt/PMMA blend from **1** was sufficiently stable to maintain the well-mixed state for >8 months at room temperature.

**Figure 4:**
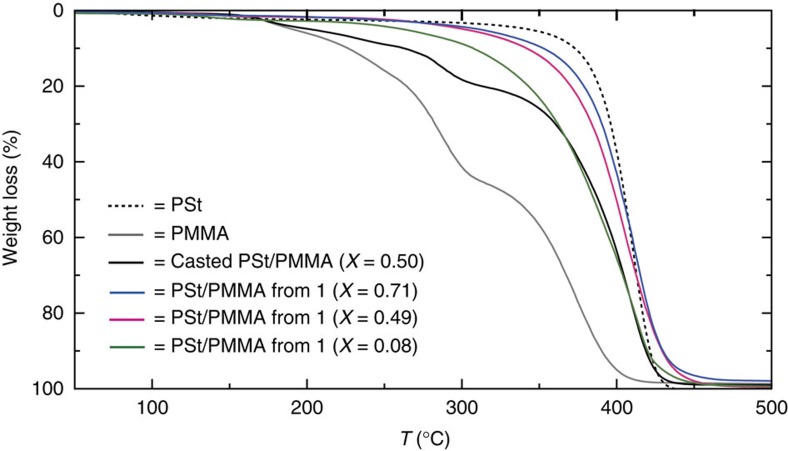
Thermal stability of polymer blends. TG curves of PSt, PMMA, cast PSt/PMMA blend and PSt/PMMA blend obtained from **1** under N_2_ atmosphere at a heating rate of 10 °C min^−1^. *X* denotes the molar ratio of PSt in the blend polymers.

The formation of polymer blends on the single nanometer scale may allow synergistic or enhanced properties that could not be achieved with the conventional physical blends. Here we studied the stability of polymer blends by thermogravimetric analysis (TGA). Figure 4 shows the degradation of PSt and PMMA homopolymers commencing at 350 °C and 180 °C, respectively, due to depolymerization of the monomers^42^,^43^. Several degradation steps were observed for the CHCl_3_-cast blend of PSt and PMMA because of the formation of their macroscopic domains^44^. In contrast, the PSt/PMMA blend prepared from **1** did not exhibit such degradation steps, but decomposed uniformly in the TGA curves as a consequence of intimate mixing. Temporary maintenance of the mixed state could be ensured by a short-time annealing experiment, where no obvious phase separation of our PSt/PMMA blend was found after a brief heat treatment above their *T*_g_s (150 °C, 1 h) (Supplementary Fig. 11). Moreover, the onset temperature of degradation of our polymer blend was *ca.* 300 °C, which is 120 °C higher than that of the cast blend, indicating a drastic increase in thermal stability of PMMA due to the molecular-level compatibilization. The improved thermal stability could be explained by effective radical quenching, in which the vicinal PSt and PMMA radicals would preferentially lead to cross-termination rather than chain transfer during the thermal degradation process^45^. The thermal behaviour of the PSt/PMMA blend could be tuned by changing the ratio of PSt in the polymer blend (*X*=[PSt]/[PSt]+[PMMA]) (Fig. 4). Interestingly, we noted a drastic increase in the thermal degradation temperature of PMMA even though the amount of PSt was very small. The degradation temperature of our polymer blend with the *X* of 0.08 was still 80 °C higher than that of neat PMMA, showing that ‘homogeneous doping' of PSt at the molecular level can significantly enhance the thermal stability of PMMA.

### Versatility of the methodology for polymer mixing

**Figure 5:**
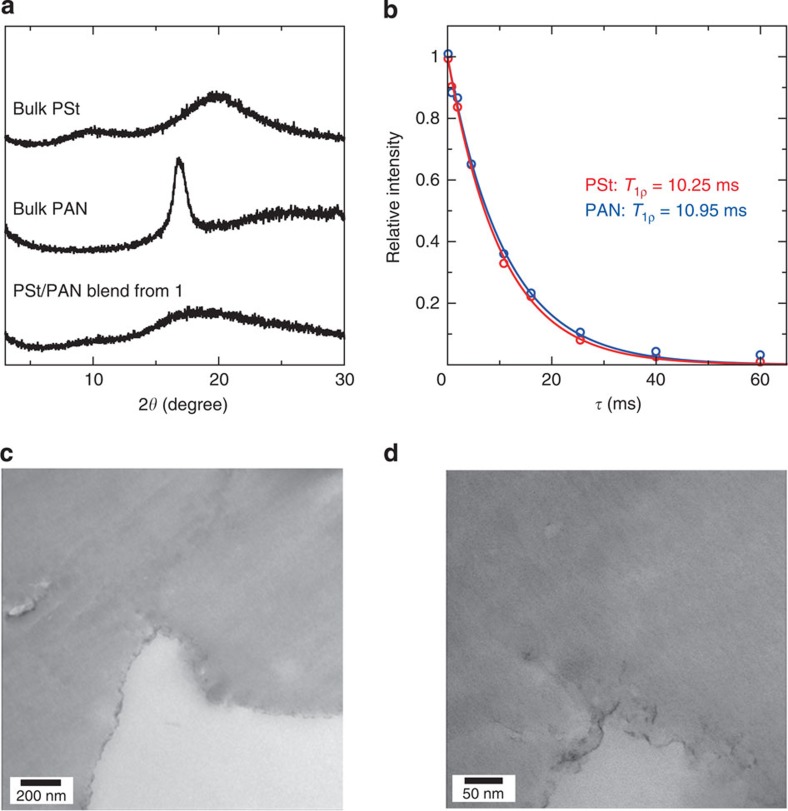
Characterizations of PSt/PAN blend obtained from 1. (**a**) XRPD patterns of bulk-synthesized PSt, PAN and PSt/PAN blend isolated from **1**⊃PSt/PAN. A characteristic diffraction peak for PAN aggregation was hardly observed in the PSt/PAN blend because of molecular dispersion of PAN in the blend. (**b**) The observed *T*_1ρ_ decay curves for PSt (red) and PAN (blue) in the polymer blend isolated from **1**⊃PSt/PAN. Almost the same *T*_1ρ_ values of PSt and PAN indicate their single nanometer level compatibilization. (**c**,**d**) TEM image of PSt/PAN blend isolated from **1**⊃PSt/PAN after staining with RuO_4_. No apparent contrast for domain structures was observed inside the polymer particles, demonstrating the complete mixing at the molecular level.

Our methodology can be applied to the intimate compatibilization of highly incompatible polymer pairs with extremely different solubility parameters. The solubility parameter of polyacrylonitrile (PAN) is 12.5∼15.4 (cal cm^−3^)^0.5^, which is quite different from that of PSt^37^. In fact, other than DMF, there are no common solvents that can dissolve both polymers. To achieve the intimate mixing of such highly conflicting polymers, we performed sequential polymerization of St and AN in **1**, followed by dissolution of the host in Na-EDTA solution (Supplementary Figs 12–16). The polymer ratio between PSt and PAN in the blend can be also determined by ^1^H NMR spectroscopy (Supplementary Fig. 15). In this system, polymerization of St in **1** was terminated after 5 h to afford an approximate 1:1 mixture. XRPD was measured to determine the miscibility of the resulting polymer blend, because PAN shows crystallinity for the hexagonal packing of the chains in the bulk state, despite its atactic stereoregularity (Fig. 5a)^46^,^47^. The diffraction peak for the PAN assembly could not be detected in the pattern of the PSt-PAN blend obtained from **1**. This result was suggestive of the molecular dispersion of PAN chains in the blend. We used solid-state NMR and electron microscopies to analyse the compatibility of the blend sample in the same way as the PSt/PMMA system. Solid-state ^13^C NMR measurement of the blend product showed that the *T*_1ρ_ values for PSt and PAN were almost the same, revealing their intimate compatibilization within the range of a few nanometer (Fig. 5b, Supplementary Figs 17 and 18). In addition, the completely homogeneous mixing of PSt and PAN in the blend sample was confirmed by TEM measurements (Fig. 5c,d and Supplementary Fig. 19). These results confirmed the high generality of our strategy, which can be extended to a wide range of immiscible polymer pairs.

## Discussion

This work established a new and viable strategy for the preparation of polymer blends mixed intimately on the single nanometer scale. A variety of immiscible and unique polymer pairs could be compatibilized by the PCP templating methodology. Due to the molecular-level compatibilization, the thermal stability of the polymer blends could be significantly improved, unlike in conventional polymer blend systems. This method would be potentially capable of scale-up production of highly compatibilized polymer blends using large amounts of PCPs. Note that the formation of polymer particles may also find attractive applications in many scientific and industrial areas. The structural and chemical diversities in PCPs allow for the rational design of polymer blends with well-defined morphology, domain size and compatibility. Moreover, PCPs are able to accommodate not only polymeric materials but also other organic molecules, biomaterials and inorganic nanoparticles, enabling the formation of functional nanohybrids for a wide range of scientific and industrial applications. We believe that this new protocol for kinetic controlled compatibilization will contribute to the creation of functional alloys and advanced nanocomposites.

## Methods

### Materials

All reagents and chemicals were obtained from commercial sources otherwise noted. The host [Zn_2_(terephthalate)_2_(triethylenediamine)]_*n*_ (**1**) was prepared by previously described methods^37^. Azobisisobutyronitrile (AIBN) was recrystallized from MeOH solution, and the vinyl monomers (St, MMA and AN) were purified by vacuum distillation prior to use.

### Preparation of polymer blends using 1

The PCP **1** (200 mg) was evacuated (<0.1 kPa) at 130 °C over 12 h, and was then immersed in a solution of freshly distilled St (1.0 ml) and AIBN (4 mg) in a flask under a nitrogen atmosphere. The mixture was left for 0.5 h to incorporate the monomer and the initiator into the nanochannels. Thereafter, excess St, external to the host compound, was completely removed under reduced pressure (0.2 kPa) at room temperature over 0.5 h. Polymerization of St in **1** was performed under nitrogen at 70 °C, over appropriate reaction times, and the reaction quenched by addition of MeOH. The resulting composite was washed with MeOH repeatedly to remove unreacted St inside the pores, followed by evacuation (<0.1 kPa) at 100 °C. Introduction of another monomer (MMA or AN) into the remaining nanochannels of **1** was performed by immersion in the monomer (2 ml) containing AIBN (4 mg), followed by evacuation of excess monomer (MMA: 1.8 kPa and AN: 8.0 kPa). Subsequently, the composite was heated at elevated temperature (MMA: 70 °C and AN: 100 °C) for 24 h to synthesize the second polymer inside the pores.

To isolate the polymers accommodated in **1**, the resulting materials were stirred in 0.5 M aqueous Na-EDTA solution for 1.5 h to decompose the framework of **1**. The collected polymer blend was washed with water and dried under reduced pressure at room temperature.

The polymer ratio in the blends could be tuned, depending on the polymerization time of St in **1**. For example, molar ratios of PSt in the blend (PSt/[PSt+PMMA]) were 0.08, 0.28, 0.49, 0.55 and 0.71 when St was polymerized for 1 h, 4 h, 6 h, 7 h and 9 h, respectively.

### Measurements

The SEM measurement was performed by use of a Hitachi S-3000 N at an accelerating voltage of 5 kV. Samples were put on a conducting carbon tape attached by SEM grid, and then coated with platinum. The XRPD data were collected on a Rigaku RINT 2000 Ultima diffractometer with Cu Kα radiation. Adsorption isotherms of nitrogen at 77 K were measured with a BELSORP mini II volumetric adsorption instrument. Nitrogen gas of high purity (99.9999%) was used. Prior to the adsorption measurements, the samples with **1** and without **1** were treated under reduced pressure (<10^–2^ Pa) for 5 h at 373 K and 298 K, respectively. The X-ray fluorescence spectroscopy was performed using Rigaku EDXL300. The infrared spectra were measured employing a Thermo Scientific Nicolet iS5. The ^1^H NMR spectra were obtained using a JEOL A-500 spectrometer operating at 500 MHz. The gel permeation chromatography measurements on the resulting polymers were performed in CHCl_3_ (for PSt and PMMA) or DMF (for PSt and PAN) at 40 °C on three linear-type PSt gel columns (Shodex K-805 L) that were connected to a Jasco PU-980 precision pump, a Jasco RI-930 refractive index detector and a Jasco UV-970 UV/vis detector set at 256 nm. The columns were calibrated against standard PSt or PMMA sample. Solid-State NMR spectra were recorded on a Bruker ADVANCE 400 MHz spectrometer. The spinning rate for all experiments was 6 kHz. ^13^C CPMAS spectra were obtained under two-pulse phase modulating proton decoupling. A recycle delay is 2 s. *T*_1_ and *T*_1ρ_ were measured at room temperature by saturation recovery method and spin lock method, respectively. The TEM measurement was performed on an electron microscope (JEM-2200FS) at an accelerating voltage of 200 kV. The instrument was equipped with a 4,096 × 4,096 slow-scan CCD camera (Gatan USC1000) and an energy filter to select transmitted and elastically scattered electrons. The PSt/PMMA and PSt/PAN samples, both from solvent casting and from polymerization inside PCP, were embedded in epoxy resin (Epok812) that were microtomed (RMC Ultracut) to thicknesses of *ca.* 100 nm and transferred on Cu grid coated with polyvinylformal (hexagonal G200HH 200 mesh). RuO_4_ vapour was used to selectively stain PSt domains after the microtoming. In some cases, the RuO_4_ staining was carried out before embedding the samples into epoxy to highlight the edge of particles from epoxy. The TGA was performed from room temperature to 500 °C at 10 K min^−1^ with a Rigaku Instrument Thermo plus TG 8120 in a nitrogen atmosphere.

## Additional information

**How to cite this article:** Uemura, T. *et al.* Mixing of immiscible polymers using nanoporous coordination templates. *Nat. Commun.* 6:7473 doi: 10.1038/ncomms8473 (2015).

## Supplementary Material

Supplementary Information Supplementary Figures 1-19

## Figures and Tables

**Figure 1:**
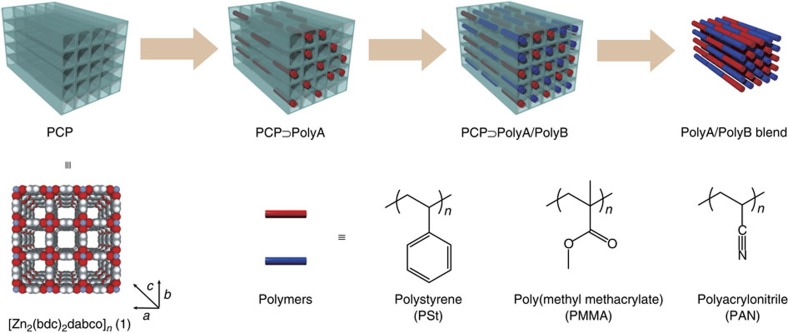
Preparation of polymer blends using PCP templates. A variety of polymer pairs can be prepared sequentially in nanochannels of **1**. Removal of the PCP host by dissolution affords polymer blends mixed intimately in the molecular level.
