# Supplementary material for: Mixing of immiscible polymers using nanoporous coordination templates
Source: Nat Commun. 2015 Jul 1;6:7473. doi: 10.1038/ncomms8473 (PMC4506999; doi:10.1038/ncomms8473)
Supplement: Supplementary Information — Supplementary Figures 1-19 [file ncomms8473-s1.pdf]

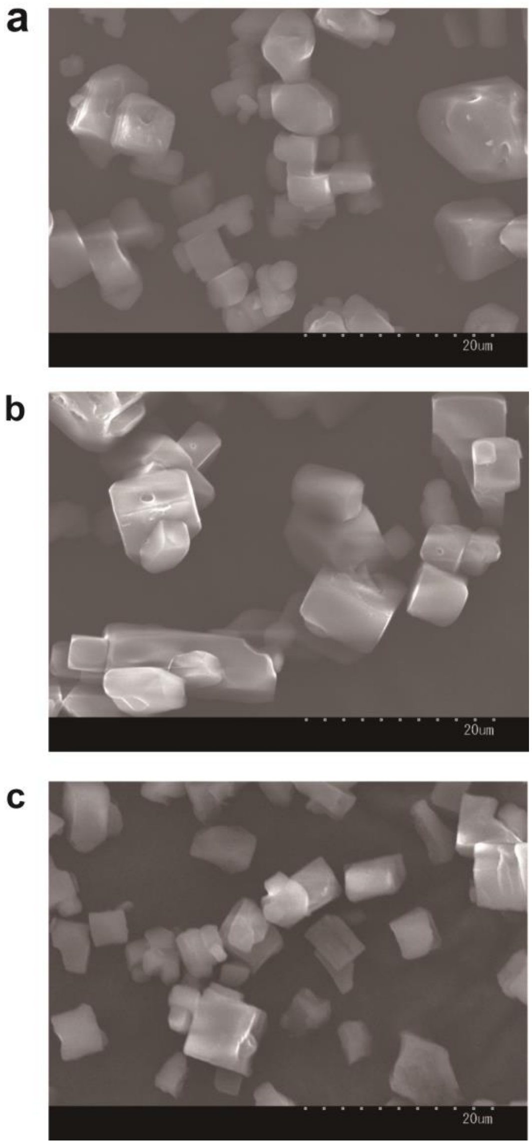

Supplementary Figure 1. SEM images of (a) **1**, (b) **1**⊃PSt/PMMA, and (c) polymer blend isolated from **1**⊃PSt/PMMA. The size and morphology of the host crystals were the almost same to those of the blend polymer particles, showing that polymerizations of St and MMA proceeded only within the pores of **1**, and polymer arrangement was not severely disturbed during the isolation process.

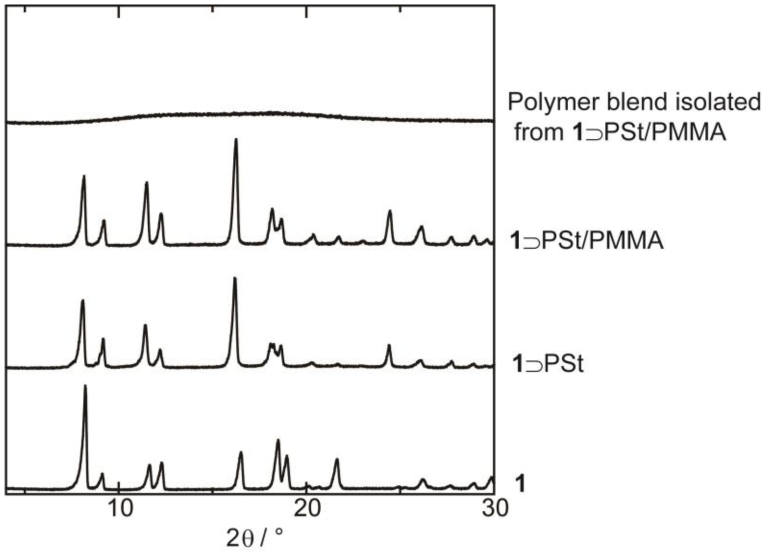

Supplementary Figure 2. XRPD patterns of **1**, **1**⊃PSt, **1**⊃PSt/PMMA, and polymer blend isolated from **1**⊃PSt/PMMA. The same diffraction patterns were observed in **1**, **1**⊃PSt, and **1**⊃PSt/PMMA indicated that the channel structure of **1** was maintained during the polymerizations of St and MMA. No peaks for **1** were detected in the diffractogram of polymer blend isolated from **1**⊃PSt/PMMA because the framework of **1** was completely removed.

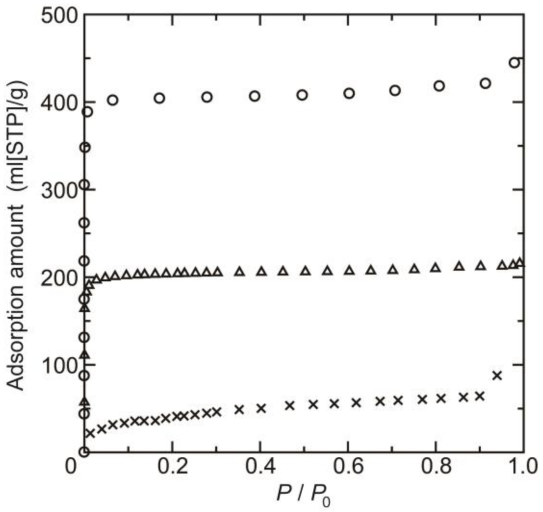

Supplementary Figure 3. N_2 adsorption isotherms of **1** (circle), **1**⊃PSt (triangle), and **1**⊃PSt/PMMA (cross) at 77 K. The adsorption isotherms **1**⊃PSt showed the decrease in the amount of adsorption compared with that of **1** because of the accommodation of PSt in **1**. Further decrease in the adsorption amount in **1**⊃PSt/PMMA clearly indicates the presence of both PSt and PMMA chains in the nanochannels.

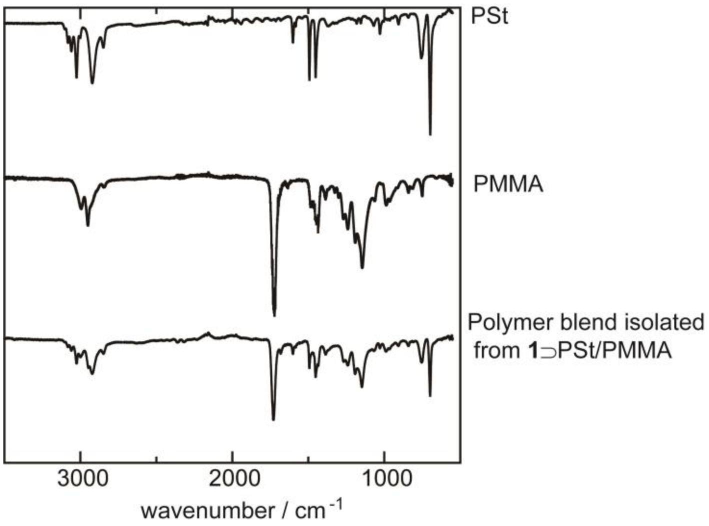

Supplementary Figure 4. IR spectra of PSt, PMMA, and polymer blend isolated from **1**⊃PSt/PMMA. IR absorption peaks for the PSt/PMMA blend were superposed on the peaks corresponding to homopolymers of PSt and PMMA.

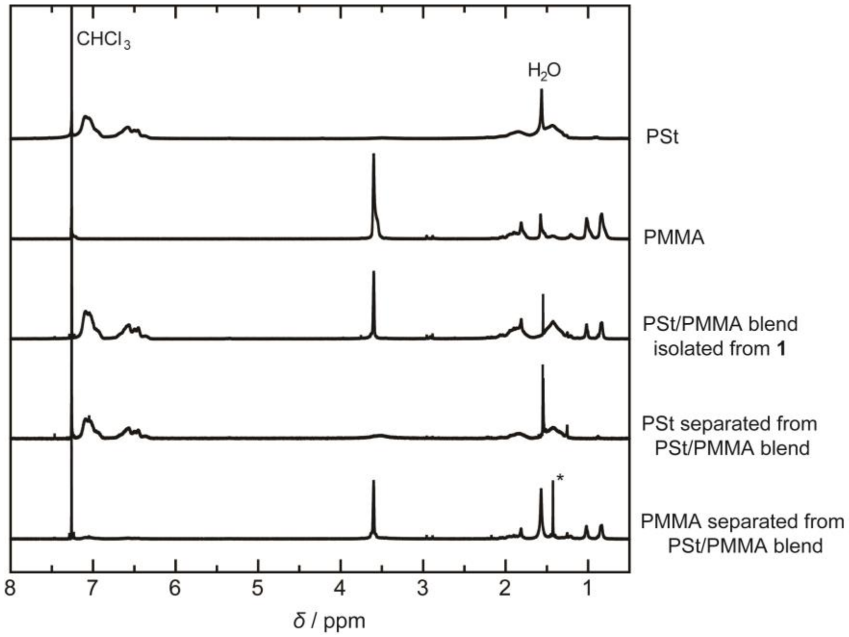

Supplementary Figure 5. ^{1}H NMR spectra of PSt, PMMA, PSt/PMMA blend isolated from **1**⊃PSt/PMMA, and homopolymers separated from the PSt/PMMA blend in $CDCl_3$ (* denotes cyclohexane). The peaks for PSt/PMMA copolymers were not detectable in the spectrum of PSt/PMMA blend isolated from **1**, indicating that the blend polymer was composed of homopolymers of PSt and PMMA. Polymer ratio in the blend can be determined by comparing the peak integration of aromatic protons (5H; 6.3~7.2 ppm) for PSt to that of methoxy protons (3H; 3.6 ppm) for PMMA in the spectrum of PSt/PMMA blend. Separation of the homopolymers was performed by pouring a $CHCl_3$ solution of the PSt/PMMA blend into cyclohexane, giving PMMA as precipitate. Evaporation of the cyclohexane solution and subsequent washing with acetic acid provided PSt. These NMR results clearly indicate that PSt/PMMA blend obtained from **1** was composed of homopolymers of PSt and PMMA.

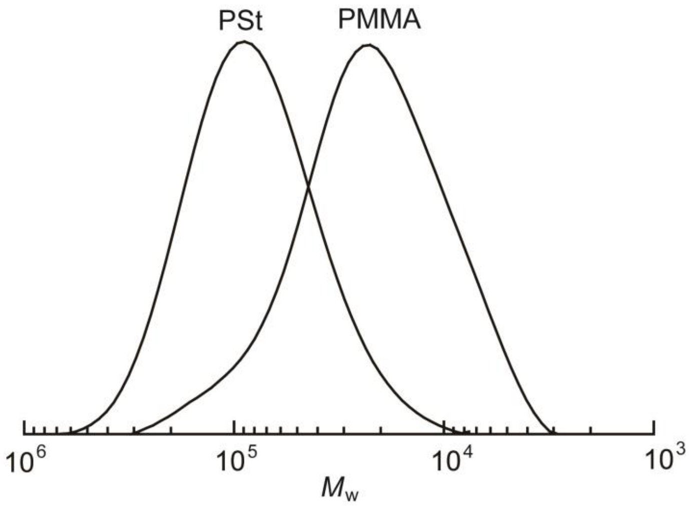

Supplementary Figure 6. Typical GPC profiles of PSt and PMMA homopolymers in $CHCl_3$ after the separation from PSt/PMMA blend prepared by using **1**. No further increase in the molecular weight of PMMA (M_n = 16,700, M_w/M_n = 1.89) in comparison to that of PSt (M_n = 63,500, M_w/M_n = 1.63) can also rule out the possibility of the formation of block copolymer.

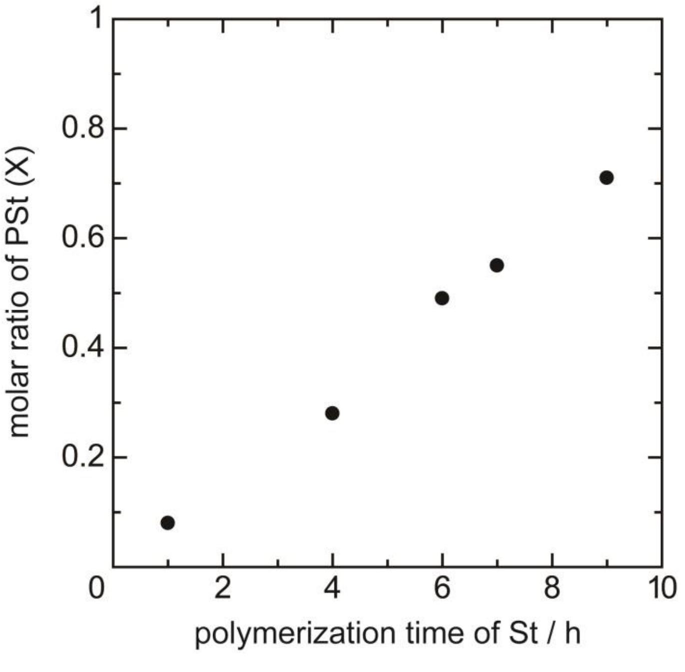

Supplementary Figure 7. Molar ratio of PSt in PSt/PMMA blend obtained from **1** (X = [PSt]/[PSt]+[PMMA]) at the polymerization time of St. The molar ratio X increased linearly with increasing the polymerization time of St.

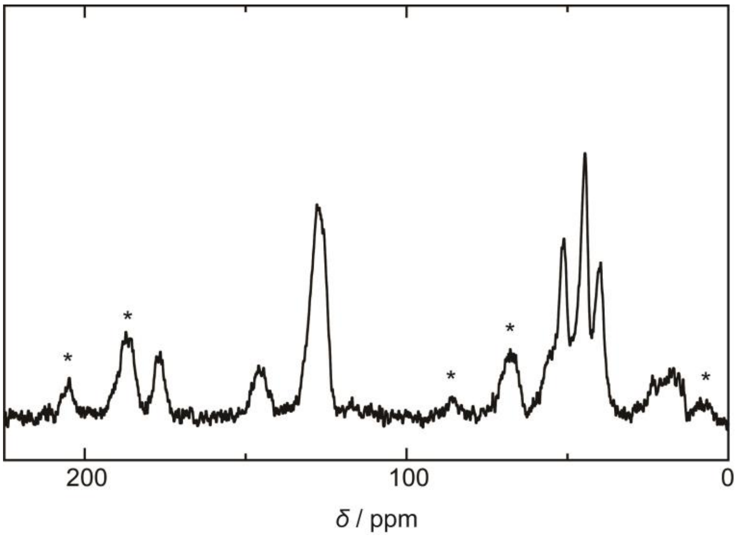

Supplementary Figure 8. Solid state ^{13}C CPMAS NMR spectrum of PSt/PMMA blend isolated from **1**. * corresponds to spinning side bands. Signals at 126 and 176 ppm assigned to phenyl moiety of PSt and carbonyl carbon of PMMA, respectively, were used for analysis of ^{1}H spin-lattice relaxation times in laboratory frame (T_1) and in rotating frame ($T_{1\rho}$). The ^{1}H relaxation times can be indirectly obtained from the ^{13}C CPMAS measurement because magnetization of protons transports to proximate carbons through ^{1}H-^{13}C dipole-dipole interaction during cross polarization. Fast ^{1}H spin diffusion through ^{1}H-^{1}H dipole-dipole interaction in a polymer results in the equal relaxation time for all protons in the polymer. Therefore, the same ^{1}H relaxation times can be obtained from any peaks of a polymer in the ^{13}C CPMAS spectrum. In the case of polymer blend, the ^{1}H spin diffusion can occur among all protons in the blended polymers, so that the ^{1}H relaxation times for the component polymers become identical. Measurement on T_1 and $T_{1\rho}$ values gives information on the formation of homogeneous domains on the scale of 20-50 nm and 2-5 nm in the polymer blend, respectively.

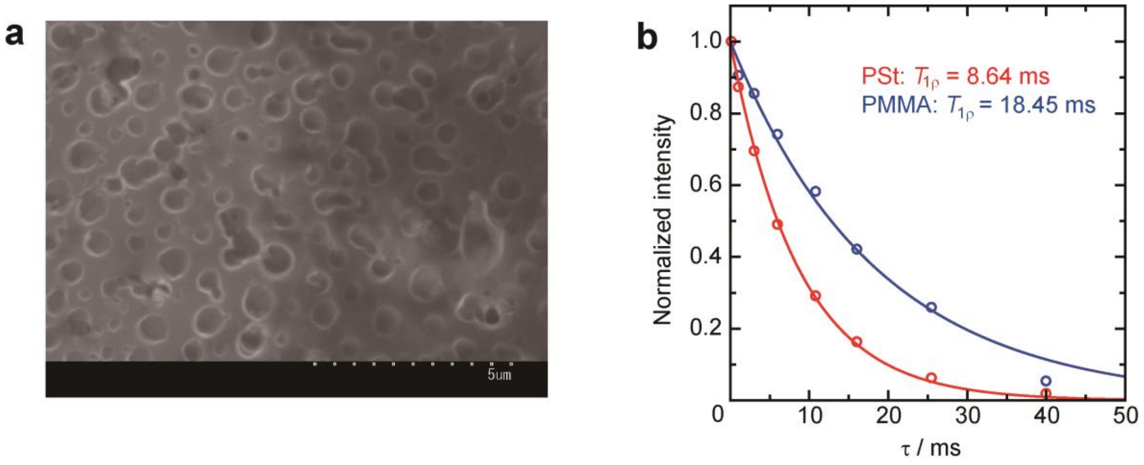

Supplementary Figure 9. (a) SEM image of the PSt/PMMA blend obtained from **1** after casting from the $CHCl_3$ solution followed by washing with cyclohexane to remove PSt domains selectively. Treatment of the polymer blend with the good solvent resulted in macroscopic phase separation. (b) The observed $T_{1\rho}$ decay curves for aromatic carbons of PSt (red) and carbonyl carbon of PMMA (blue) in the PSt/PMMA blend from **1** after casting from the $CHCl_3$ solution. Quite different $T_{1\rho}$ values for PSt and PMMA clearly supported the phase separation.

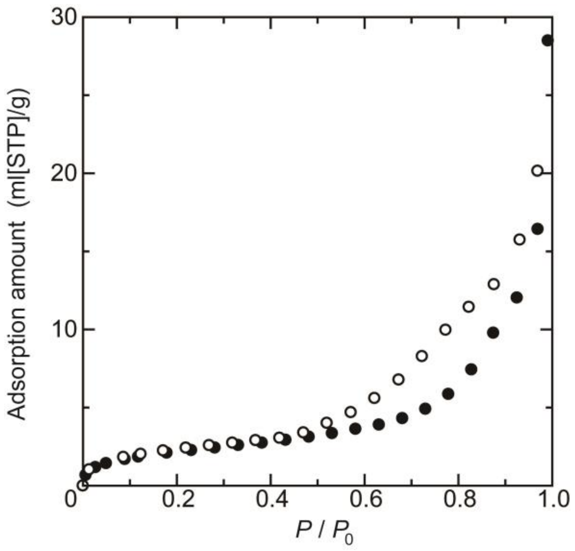

Supplementary Figure 10. N_2 adsorption (filled circle) and desorption (open circle) isotherms of PSt/PMMA blend prepared from **1** at 77 K. Although the porosity of this material is very low, type IV isotherms with the hysteresis behavior indicated the existence of meso- and macropores in the blend particles, which also contributed to compensating for the deformation of the polymer particles.

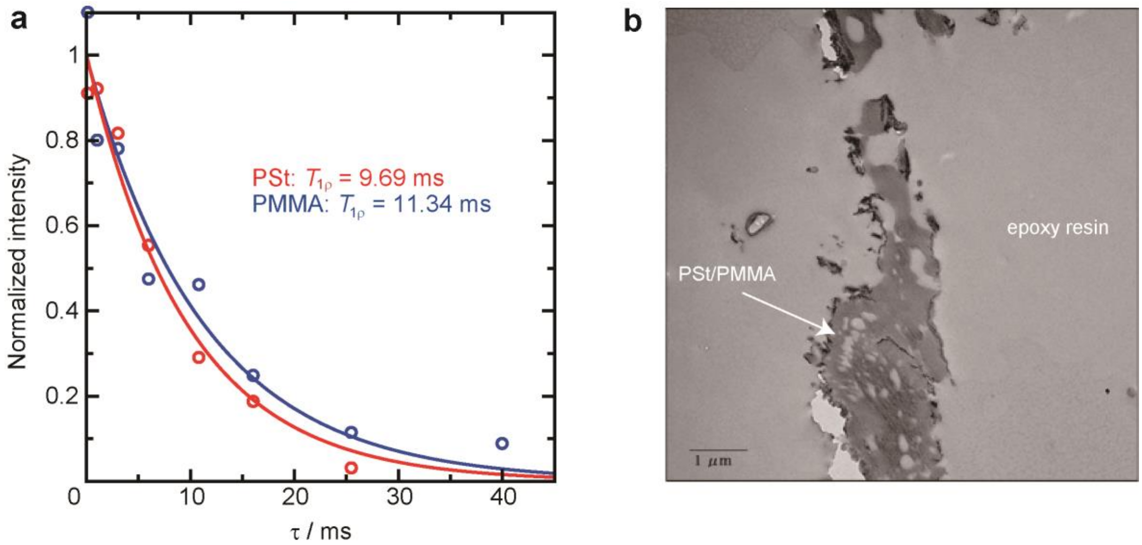

Supplementary Figure 11. (a) The observed $T_{1\rho}$ decay curves for aromatic carbons of PSt (red) and carbonyl carbon of PMMA (blue) and (b) TEM image of PSt/PMMA blend from **1** after annealing at 150 °C for 1h. (a) $T_{1\rho}$ values for PSt and PMMA was still similar, suggesting that compatibility of the polymer blend was maintained. (b) TEM of the annealed sample after staining with RuO_4 showed no obvious phase separation in the macroscopic level, although small contrasted phases with nanoscale domains were observable.

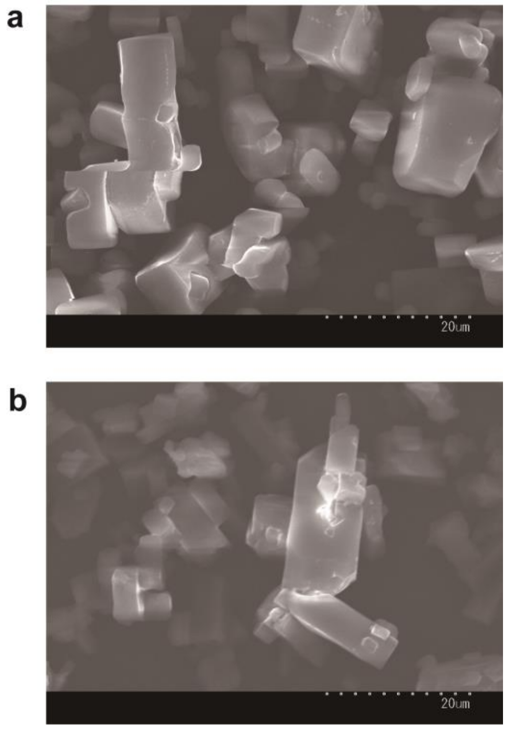

Supplementary Figure 12. SEM images of (a) **1**⊃PSt/PAN and (b) polymer blend isolated from **1**⊃PSt/PAN. As is the similar to PSt/PMMA system, morphology of the PSt/PAN blend from **1**⊃PSt/PAN are the almost identical to that of **1**⊃PSt/PAN.

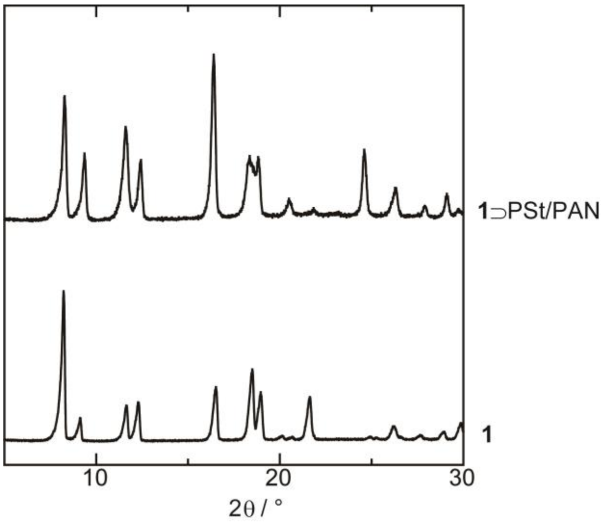

Supplementary Figure 13. XRPD patterns of **1** and **1**⊃PSt/PAN. The peak positions in the diffractogram of **1**⊃PSt/PAN were the same to those of original **1**, showing that the channel structure of **1** was maintained during the polymerizations of St and AN.

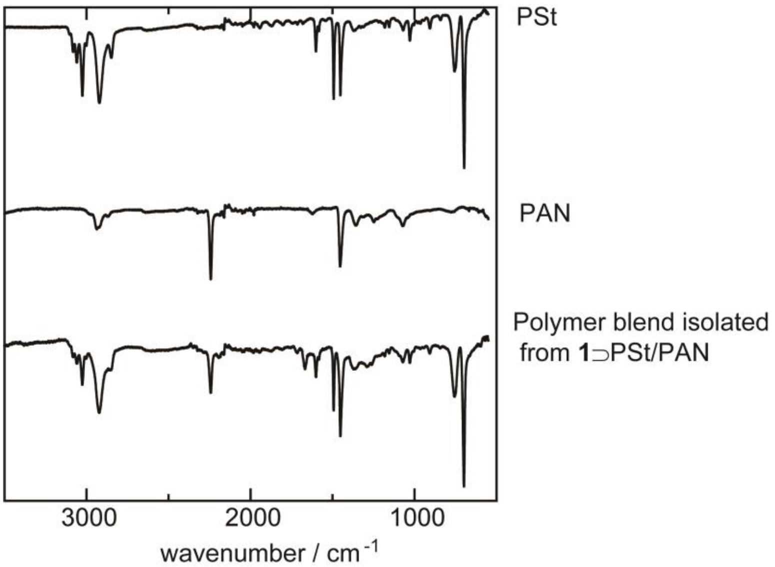

Supplementary Figure 14. IR spectra of PSt, PAN, and polymer blend isolated from **1**⊃PSt/PAN. IR absorption peaks for the PSt/PAN blend were superposed on the peaks corresponding to homopolymers of PSt and PAN.

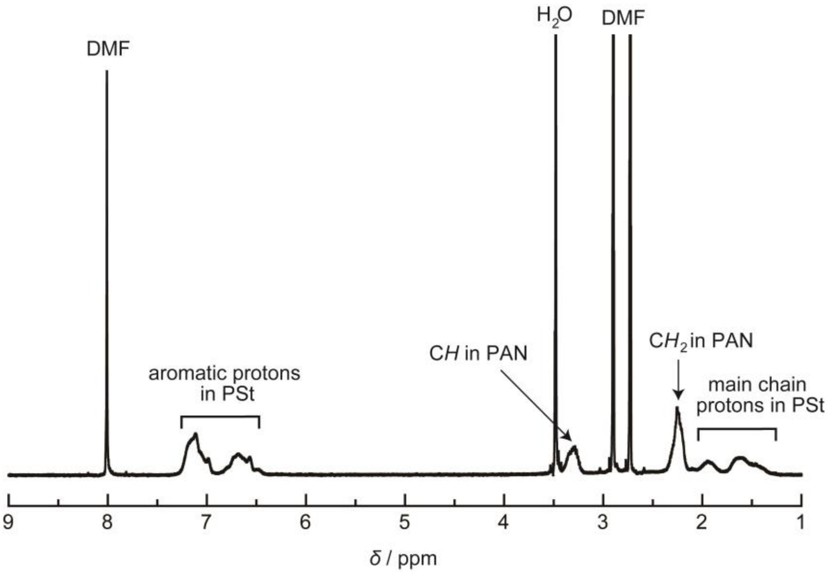

Supplementary Figure 15. ^{1}H NMR spectrum of PSt/PAN blend isolated from **1**⊃PSt/PAN in DMF-d_7. No peaks corresponding to PSt/PAN copolymers was observed. Polymer ratio in the blend can be determined by comparing the peak integration for PSt and that for PAN in the spectrum.

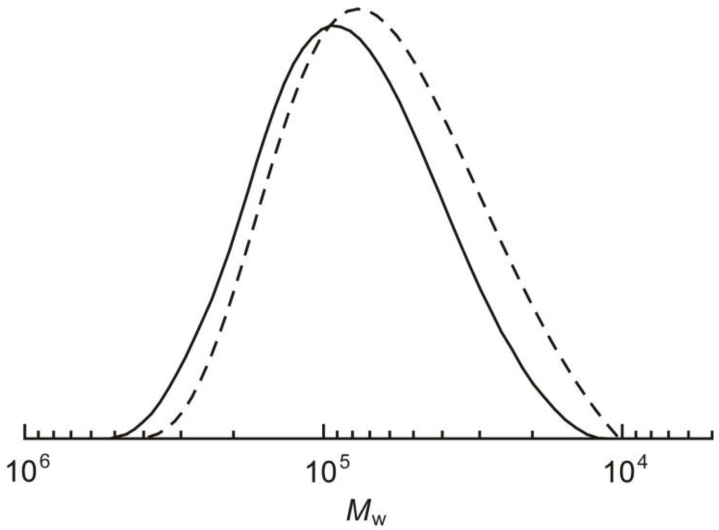

Supplementary Figure 16. Typical GPC profiles of PSt (dashed line) and PAN (solid line) homopolymers in DMF after the separation from PSt/PAN blend prepared by using **1**. Separation of the homopolymers was performed by pouring a DMF solution of the PSt/PAN blend into $CHCl_3$, which allowed to give PAN (M_n = 66,400, M_w/M_n = 1.54) as precipitate and PSt (M_n = 51,600, M_w/M_n = 1.59) in the solution.

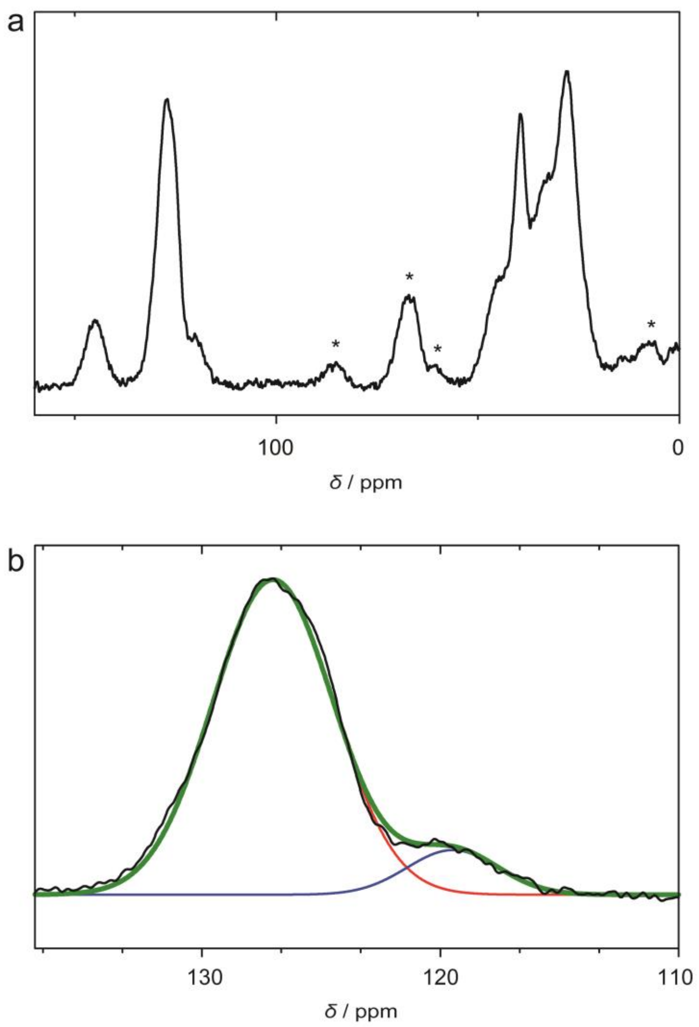

Supplementary Figure 17. (a) Extracted ^{13}C CPMAS NMR spectrum (spin lock time: 0.15 s) from the $T_{1\rho}$ measurement for PSt/PAN blend isolated from **1**. * corresponds to spinning side bands. (b) Expanded spectra of the extracted ^{13}C CPMAS NMR and simulated spectra. Overlapping peaks at 110-137.5 ppm are decomposed by two Gaussian peaks at 119.5 and 127.0 ppm. Green line represents the summation of decomposed two peaks. The decomposed peaks assigned to nitrile carbon of PAN (blue line) and phenyl carbons of PSt (red line), respectively, were used for analysis of $T_{1\rho}$.

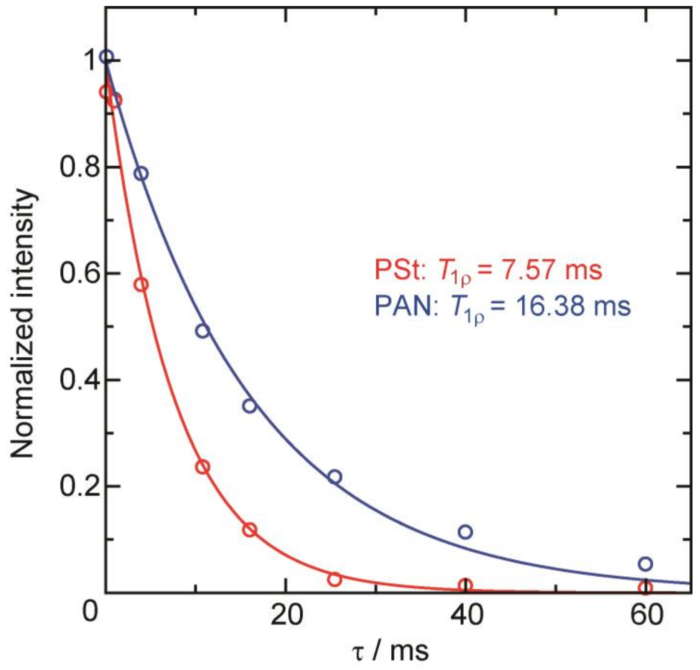

Supplementary Figure 18. The observed $T_{1\rho}$ decay curves for PSt (red) and PAN (blue) in DMF-casted blend (PSt:PAN = 1:1). Largely different $T_{1\rho}$ values for PSt and PAN ensured the clear phase separation in the this sample.

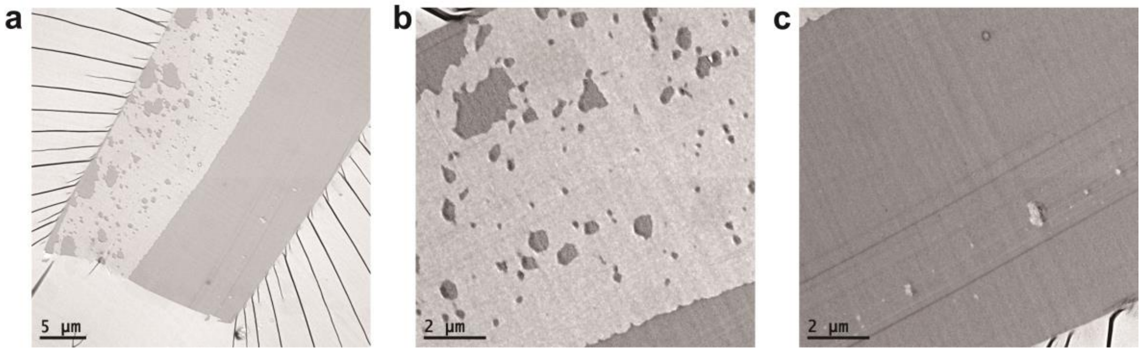

Supplementary Figure 19. TEM image of PSt/PAN blend obtained by casting from DMF solution. The sample was embedded into epoxy resin and then microtomed to thin sections, which were then stained with RuO_4 vapor to stain PSt domain. (a) Macroscopic bilayered structure with PSt and PAN rich phases was observed because of the extremely high immiscibility for these polymers. (b) Island domains of PSt were often observed in the PAN rich phase, (c) however, PAN domain was hardly detected in the PSt rich phase
